# Supplementary material for: A multi-encoder variational autoencoder controls multiple transformational features in single-cell image analysis
Source: Commun Biol. 2022 Mar 23;5:255. doi: 10.1038/s42003-022-03218-x (PMC8943013; doi:10.1038/s42003-022-03218-x)
Supplement: Supplementary file 1 — Supplementary Information [file 42003_2022_3218_MOESM1_ESM.pdf]

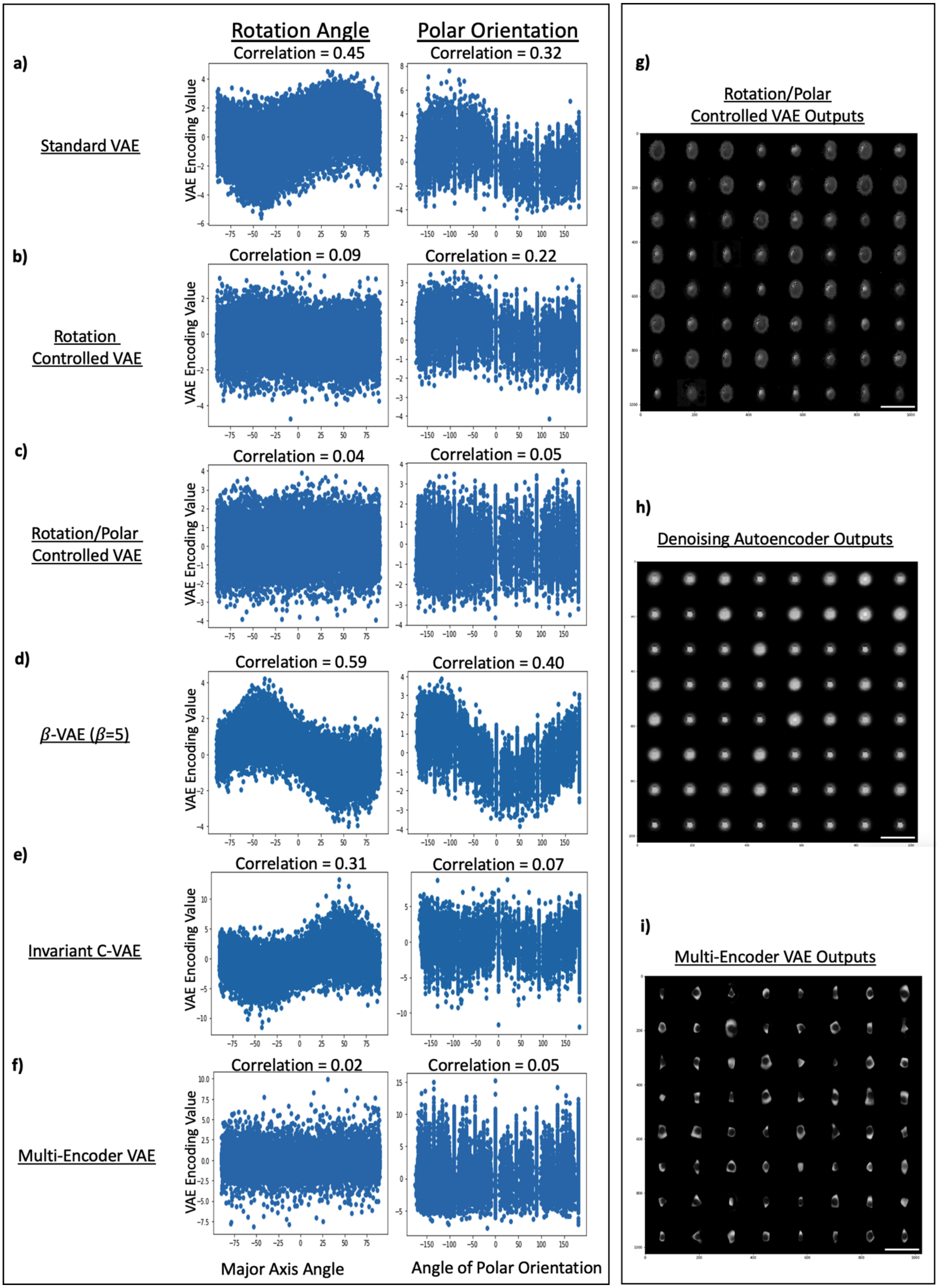

a)
b)
c)
d)
e)
f)
Rotation Angle
Polar Orientation
Correlation = 0.45
Correlation = 0.32
Standard VAE
Correlation = 0.09
Correlation = 0.22
Rotation Controlled VAE
Correlation = 0.04
Correlation = 0.05
Rotation/Polar Controlled VAE
Correlation = 0.59
Correlation = 0.40
β-VAE (β=5)
Correlation = 0.31
Correlation = 0.07
Invariant C-VAE
Correlation = 0.02
Correlation = 0.05
Multi-Encoder VAE
VAE Encoding Value
Major Axis Angle
Angle of Polar Orientation
g)
Rotation/Polar Controlled VAE Outputs
h)
Denoising Autoencoder Outputs
i)
Multi-Encoder VAE Outputs

Supplementary Figure 1: Mitigation of uninformative features for tested architectures. Encoding spaces for each VAE method were analyzed for correlation with uninformative features. Scatter plot and correlation is shown for the latent space component that had the highest correlation to given the metric. Correlations for all methods utilized a sample size of n = 15,898 single cell images **a)** Standard VAE used as baseline to show high correlation between encoded features and undesired features. **b)** Output corrected transform invariant VAE controlling for rotation only. **c)** Output corrected transform invariant VAE controlling for rotation and polar orientation. **d)** β-VAE implementing β hyperparameter in loss function. **e)** Invariant C-VAE using quantified values of uninformative features injected into decoder. **f)** Proposed multi-encoder VAE correcting for both rotation and polar orientation. **g**) Failed reconstruction examples from the transform invariant VAE correcting for both rotation and polar orientation. **h**) Failed reconstruction examples from the Denoising AE correcting for both rotation and polar orientation. **i**) Successful reconstruction examples from the ME-VAE correcting for rotation and polar orientation. Scale bars in **g-i** represent 40μm.

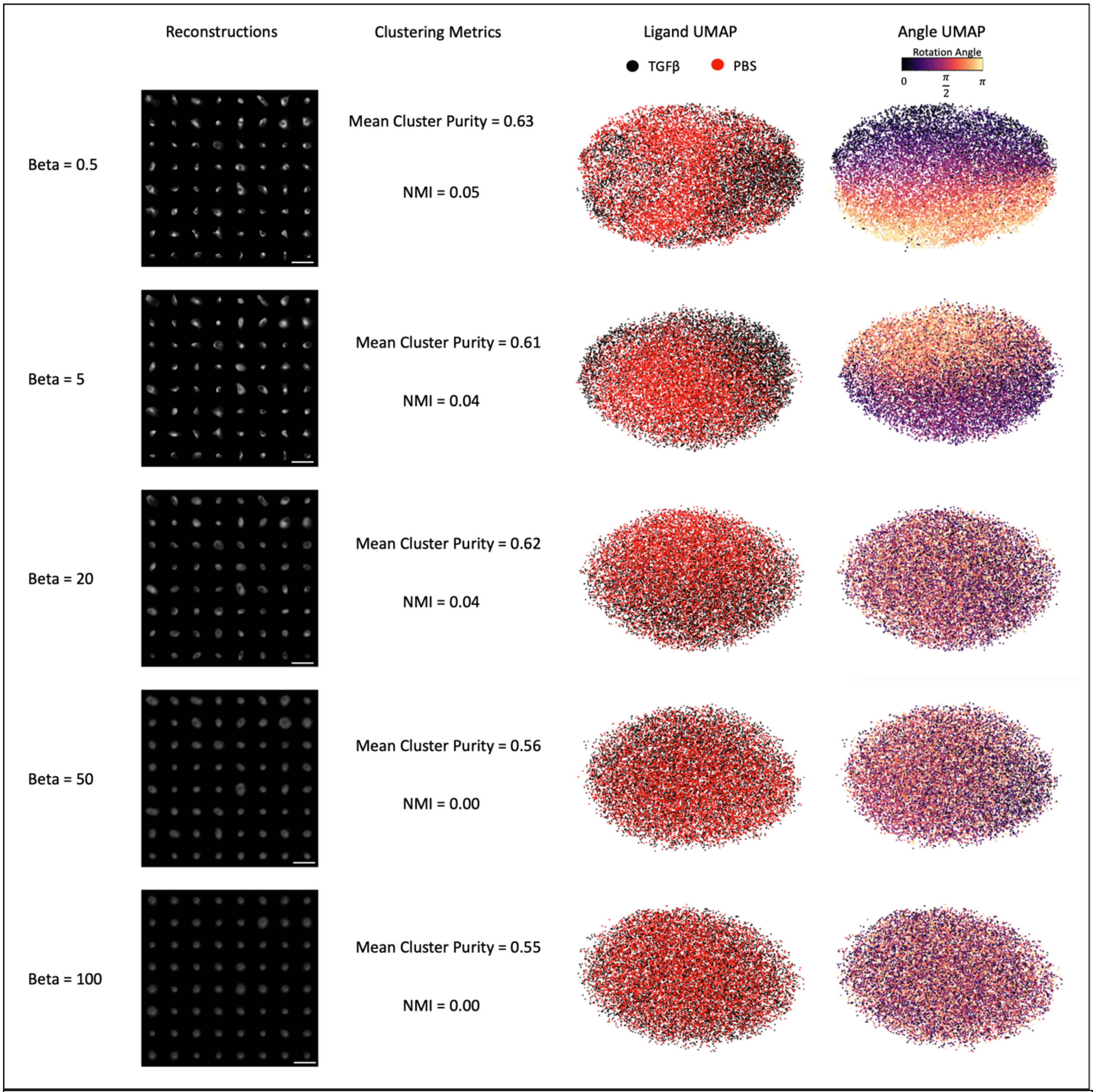

Supplementary Figure 2: Results of tuning the β hyperparameter.
Panels are shown 25 randomly sampled image reconstructions across varying values of β, followed by their quantified clustering metrics generated using k-means with number of clusters = 2 and sample size of n = 15,898 single cell images, and on the right are the models' projections into UMAP, colored by ligand population and rotation angle. Scale bars represent 40μm.

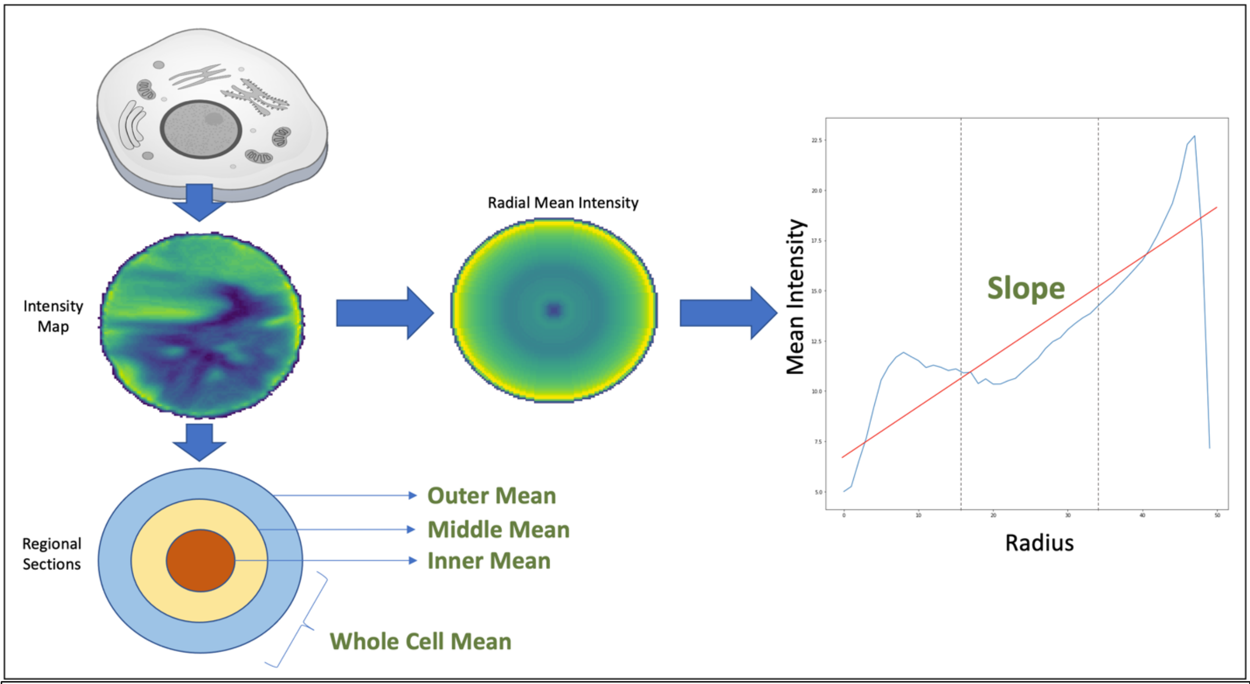

Supplementary Figure 3: Extracted biological metrics from CYCIF. Cell intensity maps were circularized to allow easy compartmentalization. The inner, middle and outer mean intensities were extracted by dividing the cell into thirds radially. The mean intensity of the whole cell was also taken. The radial mean intensity map was created by taking the average intensity for each radius across the circularized cell. The slope of the radial mean intensity map was then taken to create a single metric for stain distribution.

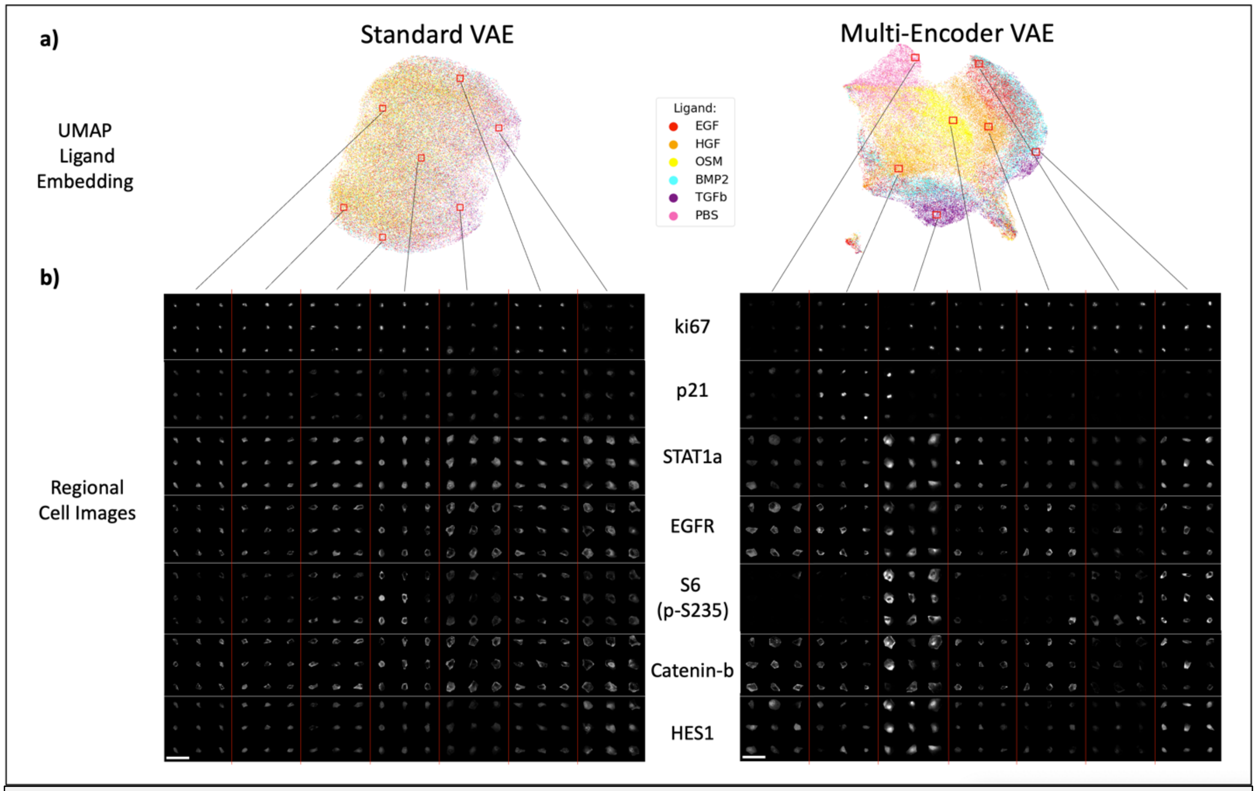

Supplementary Figure 4: Regional cell images across UMAP visualization. a) UMAP embeddings for respective VAE encodings, allowing for qualitative visual evaluation of ligand separability. **b)** Regional cell images were sampled from locations throughout UMAP space to highlight the differences in expression pattern. Stains shown were selected based on a combination of being correlated to important VAE features and hand-selection for known variance. Scale bars represent 40μm.

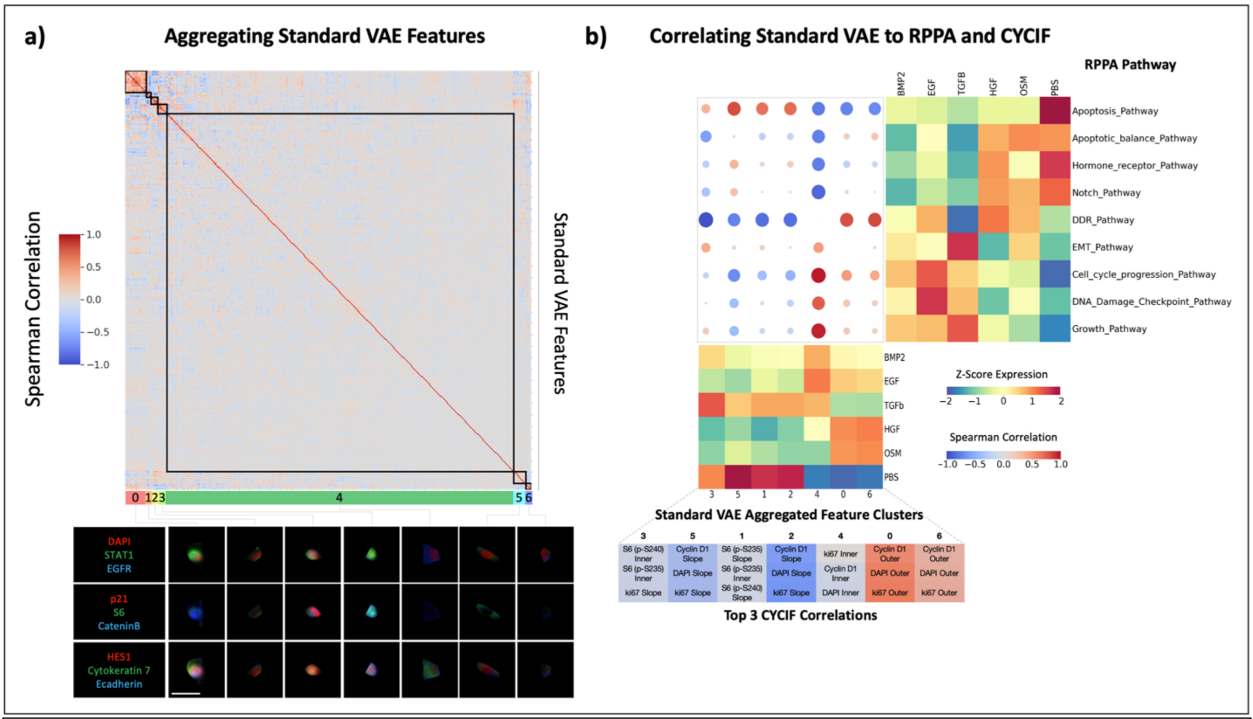

Supplementary Figure 5: Standard VAE feature aggregation and transitive inter-modality correlation.

a) Using the single cell observations as features, correlations are drawn between pairs of standard VAE features. These features are then hierarchically clustered to observe patterns and reduce VAE features to aggregated feature sets. Cell images were assigned aggregated feature scores using the mean expression of each feature in a cluster. Shown are representative cells that are highly expressing for each respective cluster. Scale bar represents 20µm for all single cell images.

b) Correlation matrix between RPPA pathway activity scores and standard VAE aggregated features. Samples from the two modalities were paired by their ligand treatments, resulting in a sample size of n=6 biologically independent ligand treated cell populations. RPPA pathways and VAE features were hierarchically clustered to show prominent patterns in correlation. Standard VAE aggregated features were also correlated to several metrics of CYCIF expression (mean inner, mean middle, whole cell means, and radial slope) for all 23 stains. This CYCIF correlation was done using the full dataset of single cell images (sample size n=73,134 single cell images)). The table of CYCIF correlations shows the top three correlations for each ME-VAE aggregated feature. Aggregated feature 4 shows high correlations to almost all RPPA pathways (3rd column from the right), and the DNA death/repair and apoptosis pathways also has high correlations to almost all aggregated features (1st and 5th rows).

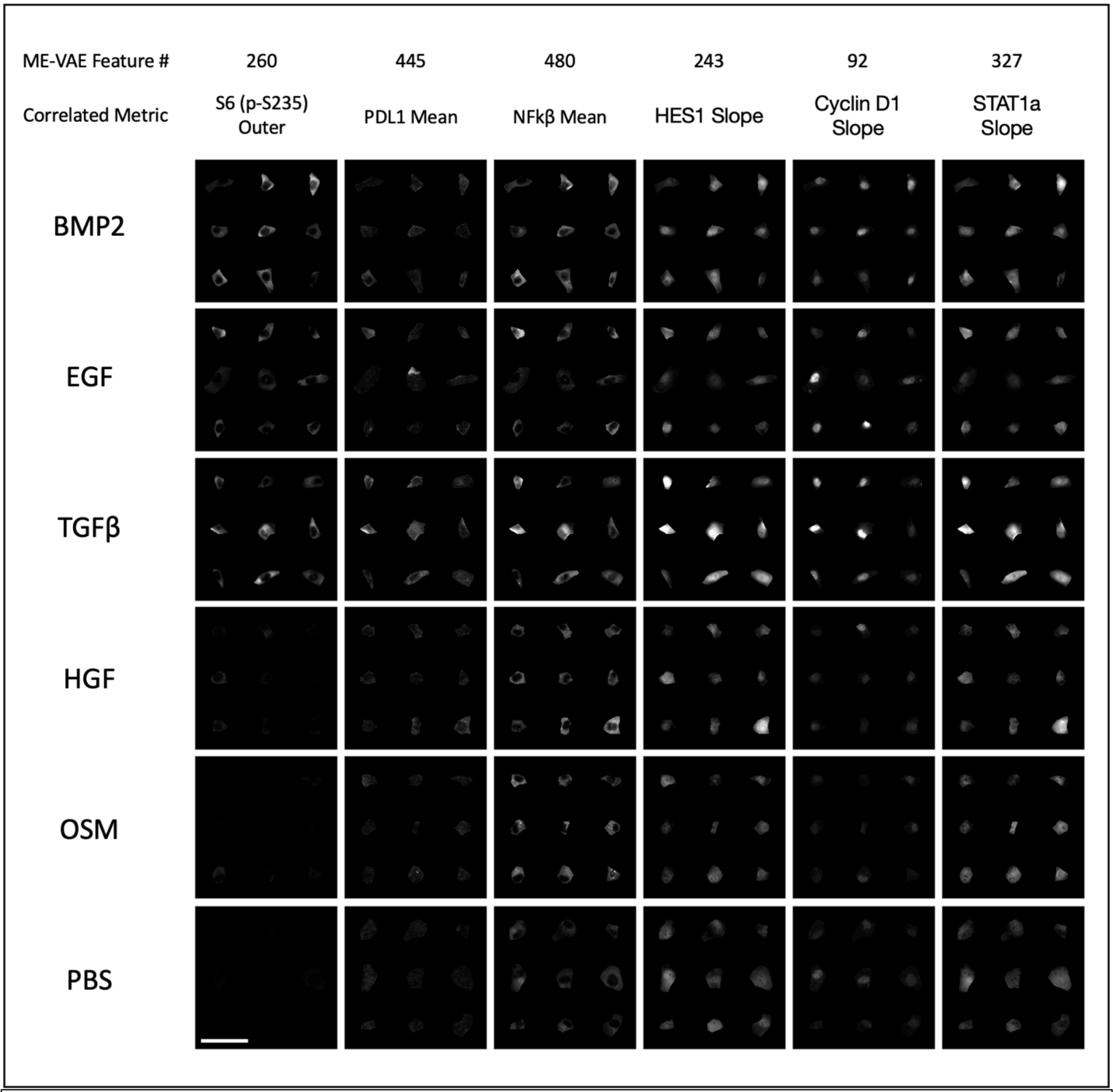

Supplementary Figure 6: Representative cell images for each ligand treatment. Representative cell images are shown for each ligand treatment (rows) and are shown using several stains (columns). Each column also includes a # that ties back to the multi-encoder feature that is highly correlated. Scale bar represents 40μm for all cell grids.

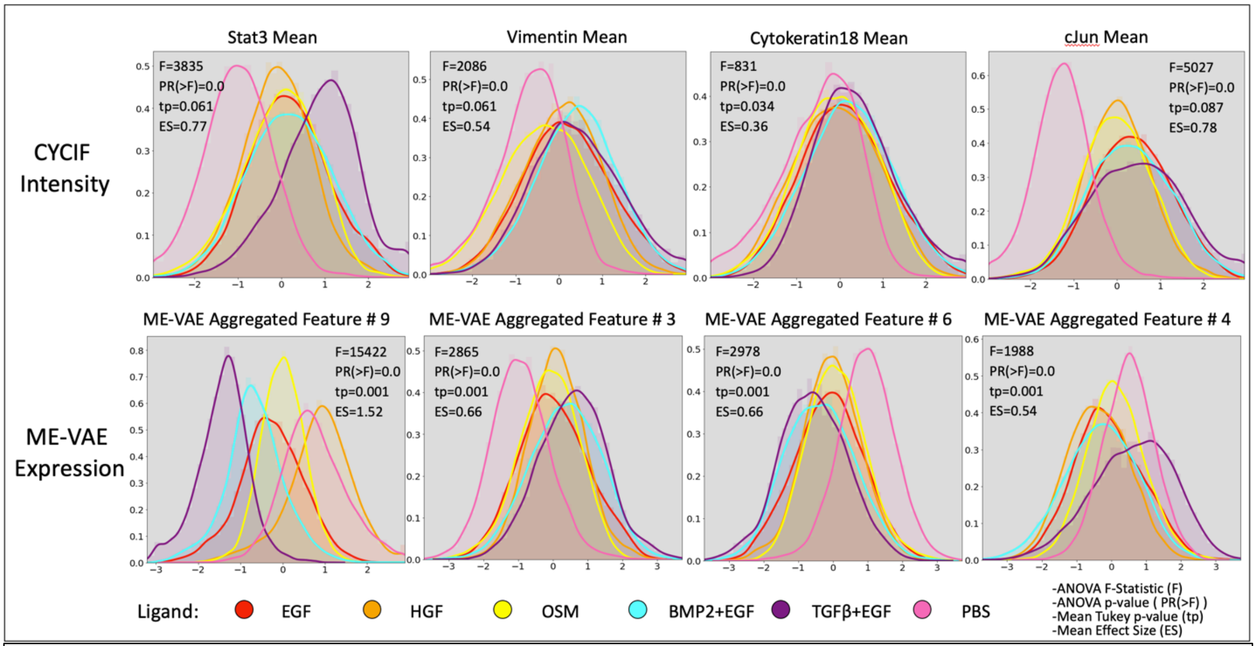

Supplementary Figure 7: Separability of ligands using aggregated ME-VAE features. Density function for several CYCIF and ME-VAE feature pairs. A two sided ANOVA was performed for a features and intensities between populations in order to compute the F statistic and p-value (PR(>F)). Subsequently, the mean Tukey-pairwise p-value across ligands and mean effect size shown for each feature. ME-VAE features used for comparison were the features with largest correlation to the respective CYCIF marker. This analysis utilized all 73,134 cell images from the MCF10A dataset.

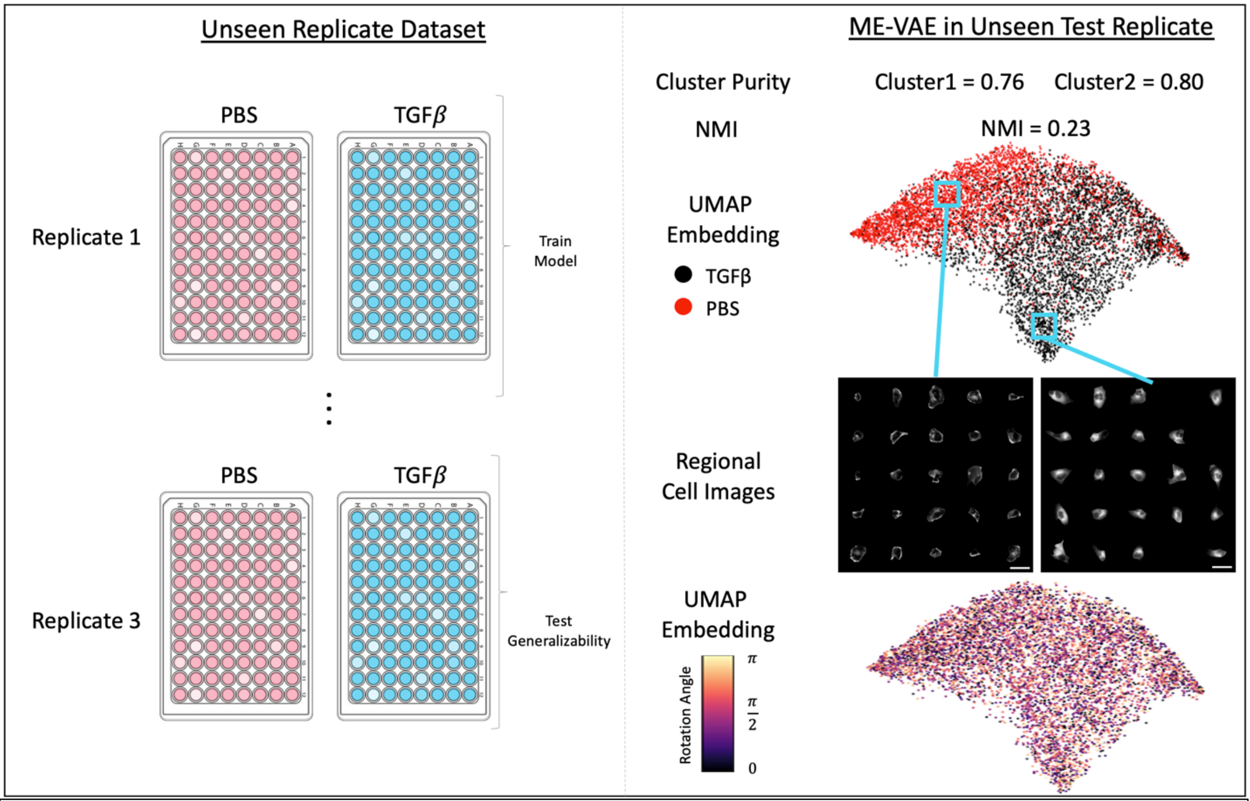

Supplementary Figure 8: ME-VAE generalizability to unseen replicate. The pre-trained ME-VAE was applied to an unseen replicate of MCF10A treated cell line data (12,229 single cell images) with the same perturbations and image normalization/augmentation steps. The model architecture is quantitatively evaluated using cluster purity and normalized mutual information (k-means with number of clusters = 2). The results of clustering (k-means with number of clusters = 2) are consistent with initial findings in the original dataset, showing good separation of labeled populations. Qualitative comparison is made using visual separation of two labeled cell populations in UMAP embedding space and visual analysis of cells from UMAP regions to identify biologically distinct factors. Rotation angle of cells are shown in UMAP embedding to show the influence of unimportant features on downstream analysis. Scale bars represent 20μm.

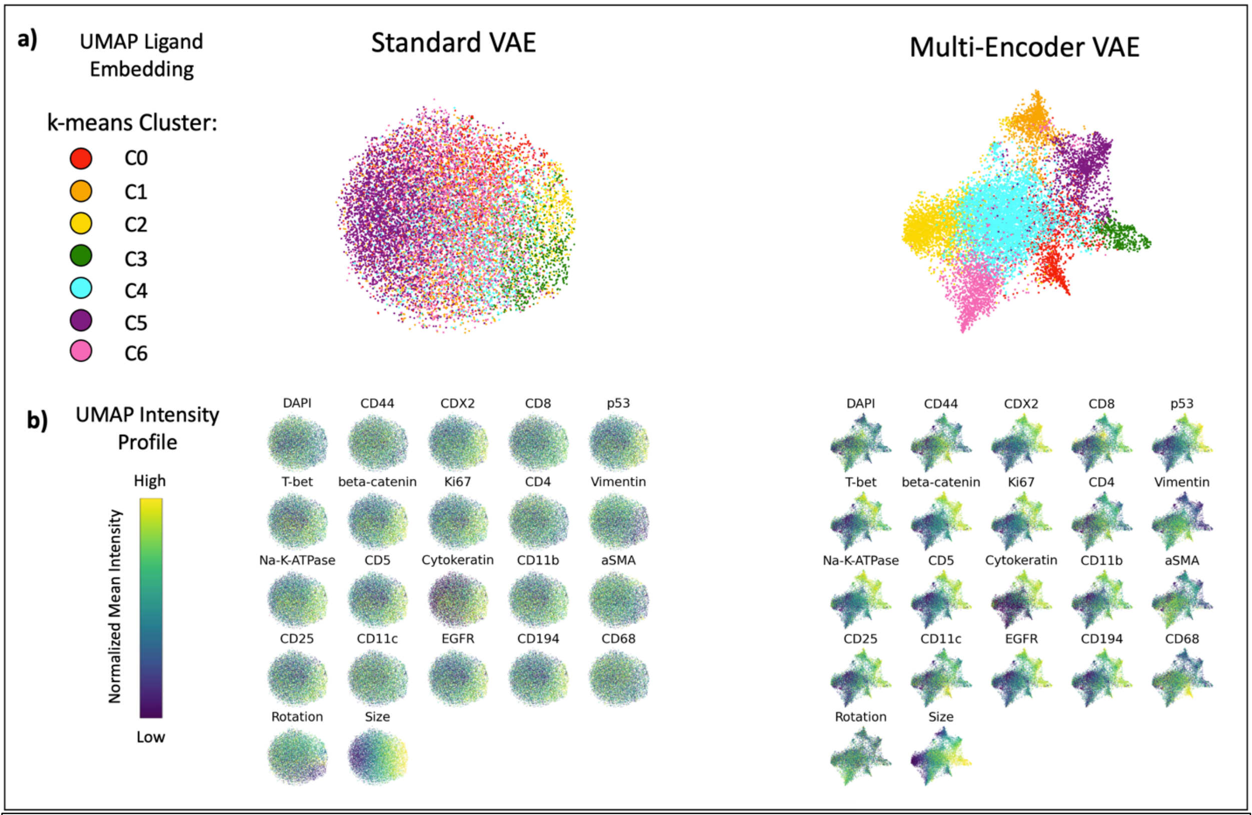

Supplementary Figure 9: Cell cluster separation and feature distribution in additional CODEX TMA dataset. a) UMAP embeddings for respective VAE encodings, allowing for qualitative visual evaluation of ligand separability. **b)** Distribution of stain features across UMAP space, colored by intensity. Both models analyzed a dataset of size n=12,229 cells individual cell images.

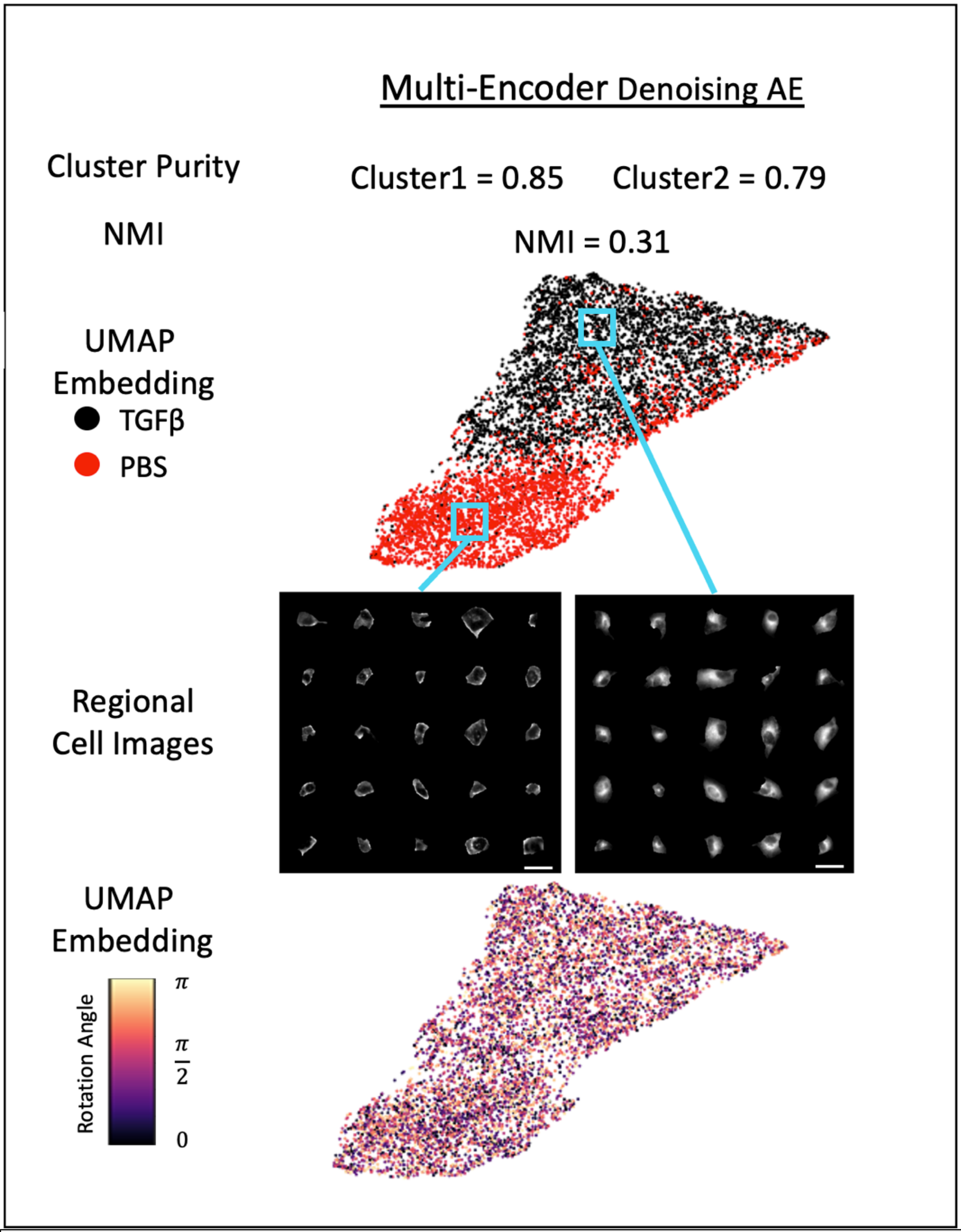

Supplementary Figure 10: Results of Multi Encoder Denoising Autoencoder. By removing the regularization term from the loss, the ME-VAE changes to use a denoising autoencoder format. The ME-DAE architecture is quantitatively evaluated using cluster purity and normalized mutual information (k-means with number of clusters = 2). The sample size is n =15,898 single cell images. Qualitative comparison is made using visual separation of two labeled cell populations in UMAP embedding space and visual analysis of cells from UMAP regions to identify biologically distinct factors. Rotation angle of cells are shown in UMAP embedding to show the influence of unimportant features on downstream analysis. Scale bars represent 20µm.

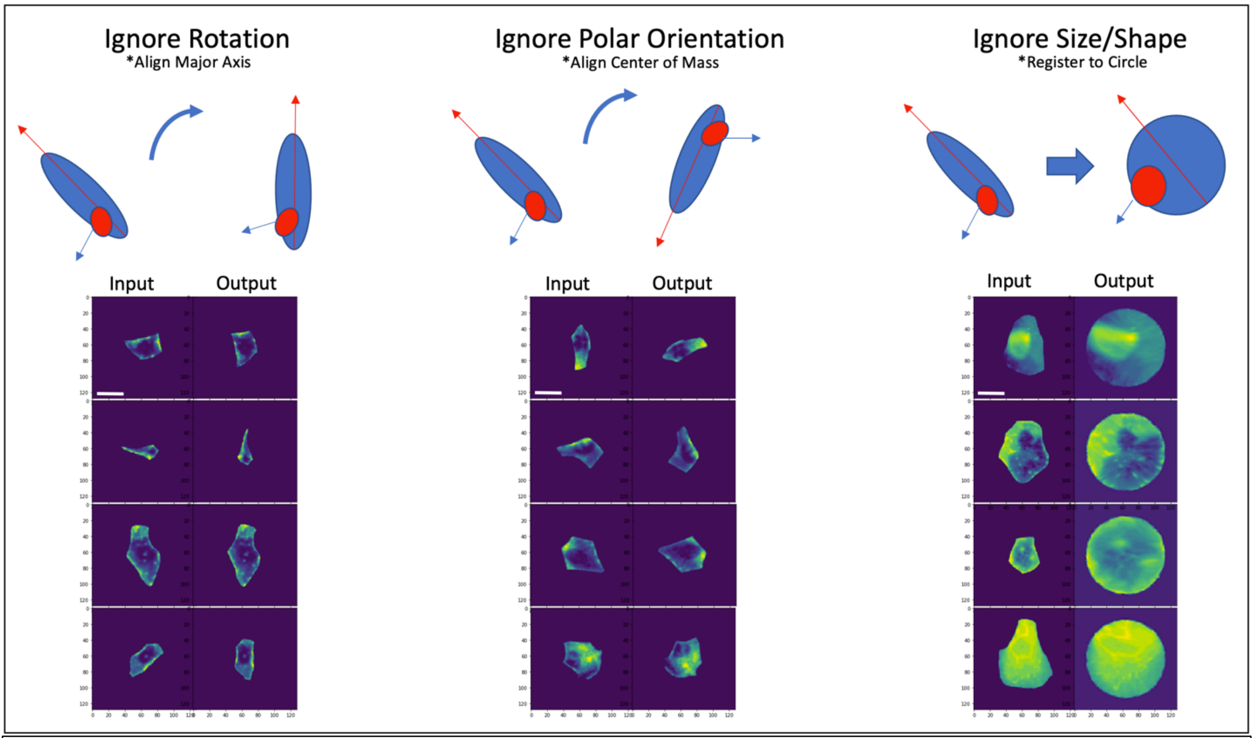

Supplementary Figure 11: Cell image transformation and correction. Examples of image corrections for rotation, polar orientation, and size/shape, shown using EGFR channel of randomly selected images. Scale bars represent 10μm for all single cell images.

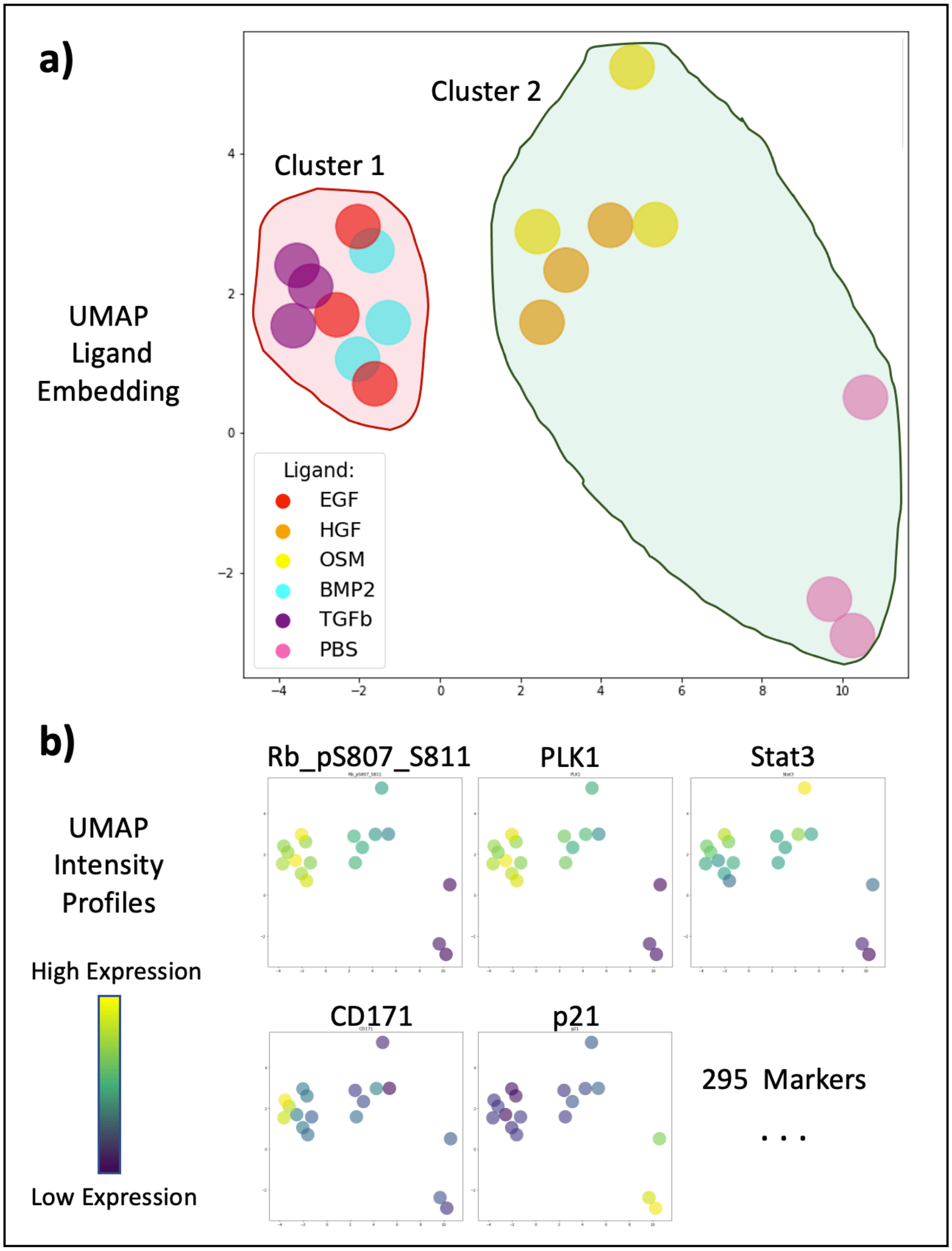

Supplementary Figure 12: Bulk RPPA analysis and clustering.
a) Independent analysis of the Bulk RPPA dataset shows distinct clustering of ligand populations in UMAP embeddings space where **b)** selected markers show clear patterns of distribution between the clusters.

Supplementary Table 1: CYCIF Marker Panel

Channel	Marker
1	DAPI
2	STAT1 (p-S727)
3	Vimentin
4	Cytokeratin 7
5	ki67
6	S6
7	LC3A/B
8	NFkB (p65)
9	p21 (Waf1/Cip1)
10	Catenin (Beta)
11	S6 (p-S235/S236)
12	PDL1
13	E-cadherin
14	STAT1 (alpha-isoform)
15	HES1
16	EGFR
17	NDG1 (p-T346)
18	STAT3
19	S6 (p-S240/244)
20	MET
21	Cytokeratin 18
22	Cyclin D1
23	c-Jun

Supplementary Table 2: RegionProps Classical Feature List

#	Property		#	Property
1	area		31	inertia_tensor-0-0
2	moments_central-0-0		32	inertia_tensor-0-1
3	moments_central-0-1		33	inertia_tensor-1-0
4	moments_central-0-2		34	inertia_tensor-1-1
5	moments_central-0-3		35	inertia_tensor_eigvals-0
6	moments_central-1-0		36	inertia_tensor_eigvals-1
7	moments_central-1-1		37	major_axis_length
8	moments_central-1-2		38	max_intensity
9	moments_central-1-3		39	mean_intensity
10	moments_central-2-0		40	minor_axis_length
11	moments_central-2-1		41	moments-0-0
12	moments_central-2-2		42	moments-0-1
13	moments_central-2-3		43	moments-0-2
14	moments_central-3-0		44	moments-0-3
15	moments_central-3-1		45	moments-1-0
16	moments_central-3-2		46	moments-1-1
17	moments_central-3-3		47	moments-1-2
18	centroid-0		48	moments-1-3
19	centroid-1		49	moments-2-0
20	eccentricity		50	moments-2-1
21	euler_number		51	moments-2-2
22	extent		52	moments-2-3
23	ferret_diameter_max		53	moments-3-0
24	moments_hu-0		54	moments-3-1
25	moments_hu-1		55	moments-3-2
26	moments_hu-2		56	moments-3-3
27	moments_hu-3		57	perimeter
28	moments_hu-4		58	solidity
29	moments_hu-5		--	--
30	moments_hu-6		--	--

Supplementary Table 3: CODEX Marker Panel

Channel	Marker
1	HOECHST
2	CD44
3	CDX2
4	CD8
5	p53
6	T-bet
7	beta-catenin
8	Ki67
9	CD4
10	Vimentin
11	Na-K-ATPase
12	CD5
13	Cytokeratin
14	CD11b
15	aSMA
16	CD25
17	CD11c
18	EGFR
19	CD194
20	CD68
