# Supplementary material for: A multi-encoder variational autoencoder controls multiple transformational features in single-cell image analysis
Source: Commun Biol. 2022 Mar 23;5:255. doi: 10.1038/s42003-022-03218-x (PMC8943013; doi:10.1038/s42003-022-03218-x)
Supplement: Supplementary file 3 — Editorial Assessment Report [file 42003_2022_3218_MOESM3_ESM.pdf]

nature portfolio GUIDED OPEN ACCESS

Contents of this report

- **Manuscript details**: overview of your manuscript and the editorial team.
- **Review synthesis**: summary of the reviewer reports provided by the editors.
- **Editorial recommendation**: personalized evaluation and recommendation from all 3 journals.
- **Annotated reviewer comments**: the referee reports with comments from the editors.
- **Open research evaluation**: advice for adhering to best reproducibility practices.

About the editorial process

Because you selected the **Nature Portfolio Guided Open Access option**, your manuscript was assessed for suitability in three of our titles publishing high-quality work across your field of research. More information about Guided Open Access can be found here.

Collaborative editorial assessment

Your editorial team discussed the manuscript to determine its suitability for the Nature Portfolio Guided OA pilot. Our assessment of your manuscript takes into account several factors, including whether the work meets the **technical standard** of the Nature Portfolio and whether the findings are of **immediate significance** to the readership of at least one of the participating journals in the Guided OA pilot.

Peer review

Experts were asked to evaluate the following aspects of your manuscript:

- **Novelty** in comparison to prior publications;
- **Likely audience** of researchers in terms of broad fields of study and size;
- **Potential impact** of the study on the immediate or wider research field;
- **Evidence** for the claims and whether additional experiments or analyses could feasibly strengthen the evidence;
- **Methodological detail** and whether the manuscript is reproducible as written;
- Appropriateness of the literature review.

Editorial evaluation of reviews

Your editorial team discussed the potential suitability of your manuscript for each of the participating journals. They then discussed the revisions necessary in order for the work to be published, keeping each journal's specific editorial criteria in mind.

Journals in the Nature portfolio will support authors wishing to transfer their reviews and (where reviewers agree) the reviewers' identities to journals outside of Springer Nature.

If you have any questions about review portability, please contact our editorial office at guidedoa@nature.com.

nature portfolio GUIDED OPEN ACCESS

Manuscript details

Tracking number		Submission date	Decision date
GUIDEDOA-21-00184		7 July 2021	8 September 2021
**Title**	ME-VAE: Multi-Encoder Variational AutoEncoder for Controlling Multiple Transformational Features in Single Cell Image Analysis	**Corresponding author**	Young Hwan Chang<br>**Affiliation:** Oregon Health & Science University
**Preprint information**	There is a preprint of this manuscript posted at: https://www.biorxiv.org/content/10.1101/2021.04.22.441005v1	**Peer review type**	Single-blind

Editorial assessment team

**Primary editor**	**George Inglis**<br>**Home Journal:** *Communications Biology*, ORCID: 0000-0002-9069-5242<br>**Email:** george.inglis@us.nature.com
**Editorial team members**	**Fernando Chirigati**, *Nature Computational Science*, ORCID: 0000-0002-9566-5835<br>**Rita Strack**, *Nature Methods*, ORCID: 0000-0003-1845-7116<br>**Kyle Legate**, *Nature Communications*, ORCID: 0000-0003-3243-579X
**About your primary editor**	George received his PhD in Genetics and Molecular Biology from Emory University, where he studied mouse models of voltage-gated sodium channel dysfunction and epilepsy. He also has research experience in epigenomics and in vitro models of neuronal development. George joined the editorial team of Communications Biology in September 2020 and is based in the New York office.

Editorial assessment and review synthesis

**Editor's summary and assessment**	Here, the authors present ME-VAE, a method that removes specified uninformative features by making them uniform and invariant across reconstructions, to improve analysis of single cell imaging data. Using CYCIF images from MCF10A cells containing various biomarkers, the authors demonstrate that ME-VAE can clearly separate a TGFβ+EGF population from controls, and outperforms standard VAE. Finally, the authors also show that there is a clear pattern of self-correlations between ME-VAE features, and identify representative clusters from these.<br>The editors decided to jointly send this manuscript out to review, given the demonstrated improvement of ME-VAE over standard VAE on multiple datasets and the potential novelty of this approach. However, *Nature Computational Science* and *Nature Methods* were concerned with the dependence of ME-VAE on prior knowledge of uninformative features and unclear generalizability and scalability of the method. The *Nature Methods* editors were also concerned about whether the method enabled new biological discovery.
**Editorial synthesis of reviews**	The reviewers seem in agreement that ME-VAE might represent an interesting method that could lead to additional practical solutions for the field, but is currently limited by the lack of benchmarking to other tools (Referees #2-3) and types of imaging data (Referees #1-2). The Referees also comment how ME-VAE's dependence on prior knowledge of uninformative transformations may limit its usage.<br>Altogether, these concerns prohibit further consideration by *Nature Computational Science* and *Nature Methods*. However, *Nature Communications* and *Communications Biology* would be interested in a revised manuscript that, at a minimum, contains the following revisions:<br>(1) As suggested by Referees #1-2, please evaluate the efficiency of ME-VAE in analyzing CODEX and/or MIBI data (per Referee #1) and normalized (vs. raw) images (per Referee #2).<br>(2) We also agree with Referees #2-3 that this method should be benchmarked to at least one other tool apart from VAE (see Referee #2's comments for specific suggestions).<br>(3) Please carefully review the GitHub code repository and include the missing information noted by Referee #3. It would also be important to clearly define the equations and variables throughout the manuscript, as highlighted by Referees #2-3.<br>(4) Given that all three referees raised concerns regarding readability, we recommend editing the main text for English language and grammar to improve readability and clarity for our readers. Please refer to the Open Research Evaluation for suggested proofreading services.

Editorial recommendation

Journal	Decision	Comments
Nature Computational Science	Revision not invited	Concerns about the conceptual and technical advance over similar methods, as well as the generalizability and scalability of ME-VAE, prohibit further consideration.
Nature Methods	Revision not invited	Given the concerns about the conceptual and technical advance over similar methods, as well as the generalizability of ME-VAE and its ability to enable new biological discovery, prohibit further consideration.
Nature Communications	Major revisions	After considering referee feedback, *Nature Communications* would require a revised manuscript to benchmark ME-VAE to additional tools, integrate new imaging datasets, and expand the analysis of ligand data as suggested by Referee #3.
Communications Biology	Major revisions	Given the referees' concerns about limited generalizability of ME-VAE, we agree it would be necessary to further benchmark this method on other imaging datasets and at least one additional tool suggested by Referee #2.

Next steps

Recommendation Summary

- **Option 1:** Revise for *Nature Communications*
- **Option 2:** Revise for *Communications Biology*

See the previous page for details. ***Nature Computational Science*** *and* ***Nature Methods*** *can no longer consider the manuscript due to concerns about the limited generalizability, biological insight, and advance over existing alternative methods.*

Revision

To follow our recommendation, please upload the revised manuscript, along with your point-by-point response to the reviewers' reports and editorial advice **using the link provided in the decision letter**. Should you need assistance with our manuscript tracking system, please contact Adam Lipkin, our Nature Portfolio Guided OA support specialist, at guidedOA@nature.com.

Revision checklist

- Cover letter, stating to which journal you are submitting
- Revised manuscript
- Point-by-point response to reviews
- Updated **Reporting Summary** and **Editorial Policy Checklist**
- Supplementary materials (if applicable)

Submission elsewhere

To a journal outside of Nature Portfolio

If you choose to submit your revised manuscript to a journal at another publisher, we can share the reviews with another journal outside of the Nature Portfolio if requested. You will need to request that the receiving journal office contacts us at guidedOA@nature.com. We have included editorial guidance below in the reviewer reports and open research evaluation to aid in revising the manuscript for publication elsewhere.

Annotated reviewer reports

The editors have included some additional comments on specific points raised by the reviewers below, to clarify requirements for publication in the recommended journal(s). However, please note that all points should be addressed in a revision, even if an editor has not specifically commented on them.

Reviewer #1	
**Reviewer #1**	This reviewer has not chosen to waive anonymity. The reviewer’s identity can only be shared with representatives of an established journal editorial office.
**Reviewer #1 expertise**	This reviewer has expertise in mathematical and computational modeling in cancer.
**Editor’s comments about this review**	This reviewer has provided an overall positive assessment of the manuscript, but notes the need for further demonstration of this method’s generalizability and scalability. In particular, they highlight the need for further benchmarking to VAE and different imaging datasets, as well as not normalizing cell size or shape. While we appreciate the reviewer's input, we must emphasize that any decisions regarding publication are made by editors. In particular, the limited generalizability of this method prohibits further consideration by *Nature Methods*. Both the limited generalizability and potential limited scalability also prohibits further consideration by *Nature Computational Science*.
**Reviewer #1 comments**	
**Overview**	The paper titled " ME-VAE: Multi-Encoder Variational AutoEncoder for Controlling Multiple Transformational Features in Single Cell Image Analysis" by Luke Ternes and colleagues describes a novel computational model called Multi-encoder VAE (ME-VAE) for single cell image feature extraction that removes specified uninformative features by making them uniform and invariant across the reconstructions, using modified pairs of transformed input and output images by self-supervised transformation, and utilizing multiple encoding blocks. Using the ME-VAE to control for these multiple transformational features, the authors are able to extract biologically meaningful and transform-invariant single cell information and better separate heterogeneous cell types. The approach is novel, aims to address an important problem, and results in improved downstream results compared to the Standard VAE using no informed transformations. The authors also illustrate the ability of ME-VAE for multi-modal integration and comparison.<br><br>I do think this is an important paper but it needs major revisions (as I detail below) and seems more appropriate for *Nature Comp Sci* or *Comms Biology*. However, if the

	authors make the changes suggested and do a great job it could be appropriate for *Nature Comms*.<br><br>There are key limitations to this work, first the lack of details pertaining to generalizability and scalability, and the reduced clarity in presentation of the data, along with incomplete explanation of figures and equations. The manuscript feels rushed and not quite ready for submission, adding to the lack or clarity and readability.

Specific comments

#	Reviewer comment	Editorial comment
**1**	The first limitation is the lack of generalizability to other emerging multiplexed technologies such as CODEX, or MIBI.	
**2**	As mentioned in the introduction, there are upcoming multiplexed imaging technologies. In the current work, the authors only show ME-VAE on CYCIF data. For generalizability of such novel methods, it is essential to demonstrate ME-VAE on one other imaging technology. There is public data available for both CODEX and MIBI.<br><br>For example see:<br>• https://portal.hubmapconsortium.org/docs/assays/codex<br>• https://www.angelolab.com/mibi-data	A revision for *Nature Communications* or *Communications Biology* should evaluate ME-VAE with at least one other imaging technology.
**3**	In the last section of Results (A), the authors mention about generalizability and scalability. To address generalizability, please refer to comment #1.	
**4**	To address scalability, please show runtime benchmarks of ME-VAE against Standard VAE for one of the experiments (e.g. between **Figure 1c-e**)	In addition to the benchmarking on alternate tools requested by Referees #2-3, both *Nature Communications* and *Communications Biology* would require a revision to evaluate runtime performance between standard VAE and ME-VAE.
**5**	Regarding known controllable transformations: The results are shown for features that are known	

	controllable transformations. These are then used as self-supervision to extract invariant features during model training. What about the case of noise-induced transformations that are unknown? Further, some of the known uninformative transformations such as rotation and polar orientation are not independent features. How do we know that these uninformative features are not getting mixed across encoders?	
6	Size and shape of a cell are important and informative features. For example, depending on the tissue being imaged and the context, certain cell types are larger than others (e.g. macrophages), or they might have a certain shape (spindle-like). This information is essential to be able to segregate them. Is it then justifiable to convert these features to being uniform and invariant across transformations?	This point would only have to be addressed as a limitation for further consideration at *Communications Biology*.
7	The crux of this work relies on transformed image pairs. What are these image pairs – an input image and its transformed output? Or are these the two transformed images, one for rotation and one for polar orientation?	
8	**Figure 2:** Legend says 'Rotation angle of cells are shown in UMAP embedding to show the influence of unimportant features on downstream analysis'. Where is this shown in the figure?	
9	**Figure 2b:** What is the input to k-means? Also mention what each dot is in the UMAP or k-means plot. How many dots are shown in the figure?	
10	What are regional cell images (e.g. in **Figure 2b-c**)? The blue square seems to have many dots whereas the zoomed in regional cell image shows 25 cells. Please also provide one higher resolution color image, with an explanation of biologically relevant features (stain localization, intensity, and subcellular pattern) within this zoomed-in regional cell image	In general, it would be necessary to provide higher-resolution images of each figure.
11	What are the radial slopes for **Figure 2c**? Since this is computed by fitting a regression line, how can a same/similar slope distinguish similar distributions for	

	different cell types?	
12	**Figure 2c:** The cluster purities from radial slope metrics, however, are still lower than the full ME-VAE cluster purity, indicating more features beyond the radial slope are being extracted from ME-VAE': Is this really a case of more features or is this a case of ME-VAE being overfit to the 'noise' that got extracted?	
13	**Figure 3:** 'Size does show some distribution in the UMAP': Please highlight this in **Figure 3, Supplementary 3b**	
14	In **Figure 4a** (bottom), each column is a cluster and is identified by a set of differentially expressed markers. Why is then each row showing a different set of differentially expressed markers per column? Same comment for **Supplementary Figure 4**	
15	Please give an example of 'morpho-spatial profiles' (mentioned in Results D)	
16	**Supplemental Figure 4b:** Please highlight or mention in the legend the row/column number where the following is observed: a 'single aggregated feature that shows significant correlations shows correlates to every RPPA pathway activity profile (Supplemental Figure 4b). Second, there is a single RPPA pathway that correlates to every standard VAE aggregated feature.'	
17	In Results D, please add citations for 'known biology', 'known literature'.	
18	In the Discussion, there is mention of 'augmenting' the model. What would an example for an augmented feature be and how would this be transformed for the ME-VAE	
19	How reliable was the EGFR channel for segmentation? For cells where the EGFR signal is not clear, would it not help to identify such cells by using additional nuclear markers for segmentation? For the extended dataset, was the segmentation again done using only	

	the EGFR channel? If only EGFR was used, why was this the case?	
**20**	**Figure 5:** 'ME-VAE features used for comparison were the features with largest correlation to the respective CYCIF marker'. Why not compare CYCIF with the ME_VAE clustered (aggregate) features? The authors already point out that they do hierarchical clustering on the ME-VAE feature 'to reduce the feature dimensionality and reduce spurious correlations in the biological findings. This comparison would also give an idea of how the clustered features look like.	
**21**	**Figure 5:** Further, how many ME-VAE and Standard VAE features were there? Is there any close correspondence between the z-scores in either column per row?	
**22**	'ME-VAE encoding features were restricted to 18 single features for each'. Does this mean that 1 ME-VAE feature = 18 single features? If this is the case, how were 18 single features assigned to one ME-VAE feature?	
**23**	**Equations in Methods B:** Please explain all the variables and what the equations do.	For the sake of reproducibility, please expand the Methods section to clearly define all variables and equations. This point has also been reiterated by Referee #2.
**24**	**Figure 1:** Mention the data used, number of cells etc. in the Figure legend.	
**25**	What are the data dimensions for the RPPA dataset?	
**26**	There are two cell numbers reported – 71314 and 73,134. Is the former after pre-processing the images?	
**27**	**C. Evaluation metrics:** Explicitly state how the slope was calculated: was it using the \beta from the regression equation?	
**28**	Which clustering method was used from the seaborn	

	clustermap function?	
29	Please spell check the document. There are typographical errors relating to words e.g. decrease, separability, reconstruction, hierarchical, python, spearman, as well as word repeats.	
30	**Supplemental Fig 4:** correct the text to reflect Standard VAE.	
31	**Figure 5:** Specify which type of ANOVA was used, and what was the p-value or F-statistic and depict this in a figure.	Please also list this information in a Statistics and Reproducibility section, as detailed in the Open Research Evaluation.
32	**Reproducibility:** The authors do host the code on GitHub and provide appropriate documentation.	

Reviewer #2

**Reviewer #2**	This reviewer has not chosen to waive anonymity. The reviewer's identity can only be shared with representatives of an established journal editorial office.
**Reviewer #2 expertise**	This reviewer has expertise in deep learning, computational biology, and computational tool development.
**Editor's comments about this review**	This referee has reiterated several of the concerns from Reviewer #1, highlighting the need for further benchmarking, and provides a valuable list of alternative methods that should be considered in a revision.

Reviewer #2 comments

**Overview**	Ternes et al. propose an extension of the classical VAE (variational autoencoder) for single cell image analysis for the purpose to extract biologically more meaningful latent representation of the input images. The main motivation is that the vanilla VAE tends to identify non-biological images features present in the dataset, such as rotation, scale etc, which can be viewed as confounding factors/ biases in the training dataset. The authors propose a method, called ME-VAE, to remove these non-informative features from the latent representation, hoping that the resulting

	new latent representation can lead to a better clustering or characterization of cell types/states.<br><br>The main idea behind ME-VAE is data normalization plus data augmentation. It generates a new set of target images that have been properly normalized, corrected based on a given set of predefined transformations. It then trains the model with random transformations of the input images, forcing the model to learn to ignore these transformations and focus on biological more meaningful features. The authors demonstrated that ME-VAE was able to yield biologically more meaningful representations than VAE through clustering and correlation analysis.

Specific comments

#	Reviewer comment	Editorial comment
**1**	It seems to me a more focused, specialized journal is more appropriate for this manuscript.	
**Major Comments:**		
**2**	The authors focus on comparing ME-VAE to vanilla VAE. However, this is highly biased for several reasons. First, there are several other recent works on single cell image analysis that have not been properly discussed, and certainly not experimentally compared. I highly recommend the authors take a close look at the methods described in the following paper and carry out a thorough comparison analysis against these existing methods.<br><br>• MCMICRO: A scalable, modular image-processing pipeline for multiplexed tissue imaging by Schapiro et al.	*Nature Communications* and *Communications Biology* would both require benchmarking to at least one additional tool.
**3**	Second, going back to the VAE method itself, it is well known that VAE does not handle confounding factors well. There are many existing works on how to correct confounding factors on VAE. Some of these methods have also been proposed for single cell genomic data analysis. A few references include:<br><br>• Moyer, D. et al. (2018) Invariant representations without adversarial training. Advances in Neural Information Processing Systems, 31, 9084–9093.<br>• Deep Generative Modeling for Single-cell Transcriptomics, Romain Lopez et al, Nature	For *Nature Communications*, comparison against one of these tools in addition to the above would be preferable, or an explanation for why they are not appropriate.

	Methods, 2018<br>• Cao et al, SAILER: Scalable and Accurate Invariant Representation Learning for Single-Cell ATAC-Seq Processing and Integration, 2021<br><br>Although they are applied to different types of datasets, the methods themselves can be applied to single cell image analysis as well. Instead of comparing with vanilla VAE, the author should compare with these more recent extensions of VAEs.	
**4**	It's also unclear to me why VAE is a good method for single cell image analysis. VAE is a generative model. The Gaussian prior applied on the latent variable tends to pull all representations toward the origin, and consequently reduces the separation between different cell types. The authors should provide a justification on why VAE is a good model for single cell analysis, and why it is better than a simpler denoise auto-encoder, the non-generative model.	
**5**	The approach works for pre-defined, well-known confounding factors such as rotation, scale. But what about latent features not associated with a well-defined transformation? It is well known that deep learning models tend to pick up correlated features that are not biologically meaningful. How do you plan to handle these features, which are a) not known beforehand, and b) may not be associated with a rigid simple transformation.	
**6**	Because the current model doesn't address batch effect, the better clustering shown in Figure 2 can potentially be associated with the batch effect. I would recommend testing the model on biological replicates of the same cell types to show that cells of the same type from different batches are mixed.	This point would be necessary for both *Nature Communications* and *Communications Biology*.
**7**	I would also like to see the results from the samples not in the training dataset. If the features are truly biologically meaningful, I would expect to see similar results on these samples as well.	
**8**	Regarding the method itself, the authors should compare with the vanilla VAE using normalized/corrected images, that is, applying VAEs on normalized images instead of raw images.	
**9**	Please use standard metrics such as ARI, NMI to evaluate clustering qualities.	

**10**	I also highly recommend the authors to test the method on a separate, ideally public dataset.	This point echoes Reviewer #1's suggestion to look at additional types of imaging data.
**Minor Comments:**		
**11**	The description of VAE models in Method B should be substantially improved. Notations are non-standard. Variables are often not defined or not referenced. Equations are unlabeled, and which loss function is for which model is not mentioned.<br>Equation of L_e seems to use terms T_i^{-1}.	
**12**	The ELBO of VAE contains a reconstruction term and a KL-divergence term encouraging smoothness of the latent space. The KL term seems to be missing from the loss functions.	
**13**	Since the vanilla VAE uses isotropic Multivariate Gaussian for prior, the KL term will facilitate different dimensions of latent z to be independent with each other. Later proposed disentanglement methods would further facilitate this independence to ensure that traversal along each dimension means interpretable data generation. This seems to be controversial to analysis in Fig. 4, where different latent features show strong correlations to each other.<br>Is there an automatic/systematic way of inferring metric for better separation of populations?	
**15**	**Line 331:** *“All models were trained for 10 epochs on the NVIDIA P100 with 100GB of memory”.*<br>Please justify 10 epochs. 100GB GPU mem is clearly incorrect.	
**16**	The main idea behind the method is straightforward. However, the code/implementation cannot be evaluated without sufficient details.<br>Github link https://github.com/GelatinFrogs/ME-350VAE_Architecture is broken.	

Reviewer #3	
**Reviewer #3**	This reviewer has not chosen to waive anonymity. The reviewer's identity can only be shared with representatives of an established journal editorial office.
**Reviewer #3 expertise**	This reviewer has expertise in computational analysis of biomedical imaging data.
**Editor's comments about this review**	This reviewer acknowledges that ME-VAE may be a first step toward improved methods for the field, but requires additional benchmarking and quantitative analyses. They also highlight the need for more detail regarding the code, to improve reproducibility.
**Reviewer #3 comments**	
**Overview**	Ternes et al. present multi-encoder variational autoencoder (ME-VAE) architecture for learning informative features from single-cell multi-channel image data. The goal is extremely significant in the field of bioimage analysis. Various approaches have been suggested during recent years to learn unbiased features instead of classical handcrafted features. These approaches enable more automated analysis solutions and importantly even robust models that can be applied to different datasets. The problem is still unsolved and the manuscript presents one possible solution. The benefit of the ME-VAE architecture presented is that it does not need any labeled data to learn the features such as in supervised learning approaches. However, ME-VAE is dependent on the knowledge of the uninformative transformations present in the data so that these can be ruled out in different encoding blocks to extract biologically informative representation in single-cell image data. Some transformations, such as rotation, are obvious, but often the challenging transformation in the data is unknown. As an example, in large datasets, experimental batch effects cause many problems for representation learning tasks (and when using classical features as well), and typically cannot be well modelled. Thus, the significance to the field is lowered in the current version of ME-VAE methodology as the users need to know these uninformative transformations present in the data. These limitations are taken into account by the authors in the discussion. I still do think this is an interesting study and could lead to more practical solutions in the future. Authors also mention in discussion that "Future applications of this architecture will allow complex features such as texture, patterns, and distribution to be extracted from single cell images without the hassle of disentangling dominant uninteresting transform features", so maybe this problem is already being studied by them.<br><br>In its current form, the most appropriate journal could be *Communications Biology*. Solving the limitations listed would improve the impact.

	The manuscript presents one approach to tackle the problem of extracting biologically meaningful unbiased features. This is an important topic in the field of bioimage analysis, especially how to learn meaningful representation without annotated data. The manuscript presents one approach to solve the problem but does not introduce novel ways of thinking in the field.

Specific comments

#	Reviewer comment	Editorial comment
**Major Comments:**		
**1**	The authors compare their ME-VAE method to standard variational autoencoder and also to variational autoencoder with corrected output. These comparisons are important to show that ME-VAE performs better than simpler VAE approaches, however, the main question should be whether ME-VAE performs better than currently used approaches. Fig 2a) includes an example of comparing two features between two ligands. Later in Fig 2d) the authors present additional feature that is inferred from visually going through the data. This single feature presented in Fig 2d gives much better separation than standard VAE approach. These classical features should be compared to standard and multi-encoder VAE to see how well existing solutions enable separation of clusters.	Building on the comments from the reviewer's overview of this manuscript, it would be useful to further highlight how this approach is distinct from previous methods in the Introduction. Furthermore, it would be necessary to compare these classical features to standard and multi-encoder VAE for consideration at both *Nature Communications* and *Communications Biology*.
**2**	As only two ligands are compared in results presented in Fig 2, I would expect the above mentioned classical feature comparison to be included also in Fig 3.	
**3**	In addition to the point made in 2. regarding Figure 3, this experiment including all 6 ligands could benefit from quantitative measurements instead of only UMAP visualization. The data could be clustered and compared using some clustering performance metric. This evaluation would quantitatively show whether the ME-VAE improves currently available methods.	Addressing this point would be necessary for further consideration at *Nature Communications*.
**Minor Comments:**		
**4**	**Line 38:** immunofluorence -> immunofluorescence	

5	**Line 120:** clusterizability and serperability: I am not sure if these are proper words	
6	**Line 170:** nucleous -> nucleus	
7	**Line 176:** unformative?	
8	**Line 331:** NVIDIA P100 with 100GB memory? Did the P100 really had 100GB GPU memory or the computer had 100GB RAM?	
9	Ref 17 is missing volume and issue information, Ref 18 is missing a title.	
10	**Line 581:** recocnstruction -> reconstruction	
11	**Fig 2d:** The cluster purity pie charts could include labels (Cluster1 left? and Cluster2 right?)	
12	**Suppl. Fig. 3 title:** "UMAP clusters" -> should replace clusters with visualization etc. as UMAP does not provide clusters, only dimensionality reduction.	
13	**Line 617:** EFGR -> EGFR	
14	**Regarding Reproducibility:** The authors share the code to train ME-VAE model, however, they do not share models trained and used to produce results in the manuscript. Or at least I was not able to find these. In addition, their code includes only an example version of ME-VAE including two parallel encoding blocks and no image data generators to prepare data for these blocks. The authors make a point that these global uninformative features are data specific which is true, but it would make reproducibility much easier by including the encoding blocks and generators used in the manuscript as an example.	For the sake of reproducibility, please include these models and image data generators along with the code.

Open research evaluation

Data availability
**Data availability statement**
Please add a Data Availability statement. Please ensure that your Data Availability statement includes accession details for deposited data, mentions where Source data can be found, and states that all other data are available from the corresponding author (or other sources, as applicable) on reasonable request.<br>• More information about our data availability policy can be found here: https://www.nature.com/nature-portfolio/editorial-policies/reporting-standards#availability-of-data<br>• See here for more information about formatting your Data Availability Statement: http://www.springernature.com/gp/authors/research-data-policy/data-availability-statements/12330880
**Code availability statement**
Please include a Code Availability statement, indicating whether and how the code can be accessed, including any restrictions to access. **In some cases, the editor may require that code be made immediately available.**<br>This section should also include information on the versions of any software used, if relevant, and any specific variables or parameters used to generate, test, or process the current dataset. **The Code Availability statement must be provided as a separate section after the Data Availability section.**<br>Please see our policy on code availability for more information: http://www.nature.com/sdata/for-authors/editorial-and-publishing-policies#code-avail<br>In addition to making the custom code available, please ensure that the version of the code/software described in the paper is **deposited in a DOI-minting repository** (eg, Zenodo) and that this DOI is also cited in the main Reference list.
**Mandatory data deposition**
For protein sequencing data, submission to a community-endorsed, public repository is mandatory for publication in a Nature Portfolio journal and is best practice for publication in any venue.<br>Accession numbers must be provided in the paper. We recommend UniProt for deposition of this data type: https://www.ebi.ac.uk/uniprot/

For proteomics data, submission to a community-endorsed, public repository is mandatory for publication in a Nature Portfolio journal and is best practice for publication in any venue. Accession numbers must be provided in the paper. Examples of appropriate public repositories are listed below:<br>• PeptideAtlas<br>• PRIDE<br>• ProteomeXchange
In general, more information on mandatory data deposition policies at the Nature Portfolio can be found at http://www.nature.com/authors/policies/availability.html#data<br>Please visit https://www.springernature.com/gp/authors/research-data-policy/repositories/12327124 for a list of approved repositories for each mandatory data type.
All **source data** underlying the graphs and charts presented in the main figures must be made available as Supplementary Data (in Excel or text format) or via a generalist repository (eg, Figshare or Dryad). This is mandatory for publication in a Nature Portfolio journal, but is also best practice for publication in any venue.
**Ethics**
Please ensure your **Competing Interests statement** includes information about all authors. Currently, competing interests for only a few authors have been declared (LT, YHC, GM, and JWG). Please note that any authors without a competing interest must also be mentioned. Furthermore, in the Editorial Policy Checklist, there is no mention of competing financial or non-financial interests.
**Reporting & reproducibility**
Please note that the information on whether the statistical test used was one-sided or two sided, where appropriate, is missing in the legend of figure 5. If space in the legends is limiting, this information can be included in a section titled “Statistics and Reproducibility” in the methods section.
Our journals strongly support public availability of data and custom code associated with the paper in a persistent repository where they can be freely and enduringly accessed or as a supplementary data file when no appropriate repository is available. If data and code can only be shared on request, please explain why in your data Availability Statement, and also in the correspondence with your editor.<br>For more information, please refer to https://www.nature.com/nature-research/editorial-policies/reporting-standards#availability-of-data

Please ensure that datasets deposited in public repositories are now publicly accessible, and that accession codes or DOI are provided in the "Data Availability" section. As long as these datasets are not public, we cannot proceed with the acceptance of your paper. For data that have been obtained from publicly available sources, please provide a URL and the specific data product name in the data availability statement. Data with a DOI should be further cited in the methods reference section.
Please ensure that all micrographs include a scale bar and this scale bar is defined on the panels or in the figure legends.
**Statistics and data presentation**
Please ensure that data presented in a plot, chart or other visual representation format shows data distribution clearly (e.g. dot plots, box-and-whisker plots). When using bar charts, please overlay the corresponding data points (as dot plots) whenever possible and always for n ≤ 10. (Please see the following editorial for the rationale behind this request and an example https://www.nature.com/articles/s41551-017-0079).
Wherever statistics have been derived (e.g. error bars, box plots, statistical significance) the legend needs to provide and define the n number (i.e. the sample size used to derive statistics) as a precise value (not a range), using the wording “n=X biologically independent samples/animals/cells/independent experiments/n= X cells examined over Y independent experiments” etc. as applicable.<br>• Although ‘n’ is provided, please describe the nature of entity for ‘n’ in the legends of **Figures 2b, 2c; 4b and Supplementary Figure 4b**.
Statistics such as error bars, significance and p values cannot be derived from n<3 and must be removed from all such cases.
We strongly discourage deriving statistics from technical replicates, unless there is a clear scientific justification for why providing this information is important. Conflating technical and biological variability, e.g., by pooling technically replicates samples across independent experiments is strongly discouraged. (For examples of expected description of statistics in figure legends, please see the following https://www.nature.com/articles/s41467-019-11636-5 or https://www.nature.com/articles/s41467-019-11510-4).
The figure legends must indicate the statistical test used. Where appropriate, please indicate in the figure legends whether the statistical tests were one-sided or two-sided and whether adjustments were made for multiple comparisons.<br>• Please note that the information on whether the statistical test used was one-sided or two-sided, where appropriate, is missing in the legend of **Figure 5**.

For null hypothesis testing, please indicate the test statistic (e.g. F, t, r) with confidence intervals, effect sizes, degrees of freedom and P values noted. Please provide the test results (e.g. P values) as exact values whenever possible and with confidence intervals noted.
Please note that the individual figure legends are not labelled as "a" and "b" for **Supplementary Figure 3**. Please rectify this in the figure legend.
**Language editing**
We recommend editing the main text for English language and grammar to improve readability and clarity for our readers. If you would like the assistance of paid editing services to do this, we can recommend our affiliates, Nature Research Editing Service:<br>Springer Nature Editing Service: https://secure.authorservices.springernature.com/en/researcher/submit/upload<br>American Journal Experts: https://www.aje.com/go/natureresearch/
**Other notes**
**Your manuscript would benefit from using fewer abbreviations**, which will help the reader keep track of the terms. Please avoid abbreviating terms unless they are used five or more times. We ask that you avoid all non-standard 2 letter abbreviations.
We have included as an attachment to the decision letter a version of your **Reporting Summary** with a few notes. This is mainly for your information, but we hope it is helpful when preparing your revised manuscript. If you decide to resubmit the manuscript for further consideration, please be sure to include an updated Reporting Summary.
