# Supplementary material for: A multi-encoder variational autoencoder controls multiple transformational features in single-cell image analysis
Source: Commun Biol. 2022 Mar 23;5:255. doi: 10.1038/s42003-022-03218-x (PMC8943013; doi:10.1038/s42003-022-03218-x)
Supplement: Supplementary file 4 — Description of Additional Supplementary Files [file 42003_2022_3218_MOESM4_ESM.pdf]

Description of Additional Supplementary Files

File Name: Supplementary Data 1
Description: EGFR intensity, cell size features for Figure 2a and EGFR Radial slope features for Figure 2h
